# CERG: Chinese Emotional Response Generator with Retrieval Method

**DOI:** 10.34133/2020/2616410

**Published:** 2020-09-07

**Authors:** Yangyang Zhou, Fuji Ren

**Affiliations:** Faculty of Engineer, University of Tokushima, Tokushima 770-8506, Japan

## Abstract

The dialogue system has always been one of the important topics in the domain of artificial intelligence. So far, most of the mature dialogue systems are task-oriented based, while non-task-oriented dialogue systems still have a lot of room for improvement. We propose a data-driven non-task-oriented dialogue generator “CERG” based on neural networks. This model has the emotion recognition capability and can generate corresponding responses. The data set we adopt comes from the NTCIR-14 STC-3 CECG subtask, which contains more than 1.7 million Chinese Weibo post-response pairs and 6 emotion categories. We try to concatenate the post and the response with the emotion, then mask the response part of the input text character by character to emulate the encoder-decoder framework. We use the improved transformer blocks as the core to build the model and add regularization methods to alleviate the problems of overcorrection and exposure bias. We introduce the retrieval method to the inference process to improve the semantic relevance of generated responses. The results of the manual evaluation show that our proposed model can make different responses to different emotions to improve the human-computer interaction experience. This model can be applied to lots of domains, such as automatic reply robots of social application.

## 1. Introduction

The dialogue system has been receiving much attention since the Turing test [1] was proposed. The dialogue system responds to the topics or instructions thrown by the user by simulating human beings [2]. Based on whether the dialogue system can achieve a specific goal, it can be divided into 2 types: task-oriented and non-task-oriented dialogue systems (or chatbot) [3]. Task-oriented dialogue systems are generally used in closed domains like ticket purchase, ordering, and customer service [4]. There are 2 main types of task-oriented dialogue systems: pipeline-based and end-to-end methods. A chatbot is generally used in open domains such as psychotherapy applications [5]. There are 3 main types of chatbot: rule-based, retrieval-based, and generation-based methods. So far, due to the application of slot filling [6] and other technologies, the task-oriented dialogue system is more mature than the chatbot. With the continuous advancement of big data and deep learning technologies, we can build a data-driven chatbot [7]. The Chinese Weibo involved in this article can be regarded as some non-task-oriented dialogue. Existing data-driven non-task-oriented dialogue systems tend to generate a safe and commonplace response [8], for example, “I don't know.” We introduce the retrieval method into the non-task-oriented dialogue system, aiming to alleviate this problem.

Dialogue generation is closely related to the technology of natural language generation. Natural language generation is a process that transforms structured data into natural language. In the domain of deep learning, the sequence-to-sequence (seq2seq) framework [9] is often used in dialogue generation. This framework consists of an encoder and a decoder, which is a kind of end-to-end learning algorithm. The encoder of seq2seq converts the input sequence into a hidden state vector. The decoder converts the vector into an output sequence, then adopts the output of the previous step as the input of the next step. With the increase of sequence length, the problem of gradient disappearance may appear in the calculation. Seq2seq avoids this problem by using long short-term memory [10] instead of original recurrent neural networks. Because the recurrent neural network cannot do the parallel calculation, the training speed is slow. The transformer model [11] proposed by Google Brain parallelizes this calculation process by the multihead self-attention mechanism, which greatly improves the calculation efficiency. Thus it has become the most commonly used model in the seq2seq framework in recent years. There is some work dedicated to improving the accuracy of translations or the quality of generated sentences. Some researchers are committed to improving the accuracy of translations [12] or the quality of generated sentences by disrupting parallel computing. We try to figure out a method to improve the quality of generated responses without disrupting parallel computing.

The key to improving the human-computer interaction experience is to make the dialogue system empathetic. Affective computing [13] is the study that can recognize and simulate human affects. Affective computing can improve the user-friendliness of the system [14]. Lots of scholars research dialogue system and affective computing, respectively. Few studies [15, 16] have linked these two aspects. Different emotions used in the same sentence usually express different meanings. This is one of the difficulties of natural language processing technology. Chinese Weibo emotional response is a task to study how to properly combine affective computing to a chatbot. The data set we adopt is from the NTCIR-14 STC-3 CECG subtask [17], which is constructed from Chinese Weibo posts and replies. This data set contains 6 different emotions: like, sadness, disgust, anger, happiness, and other. We aim to find a way to incorporate affective computing into dialogue generation.

**Figure 1:**
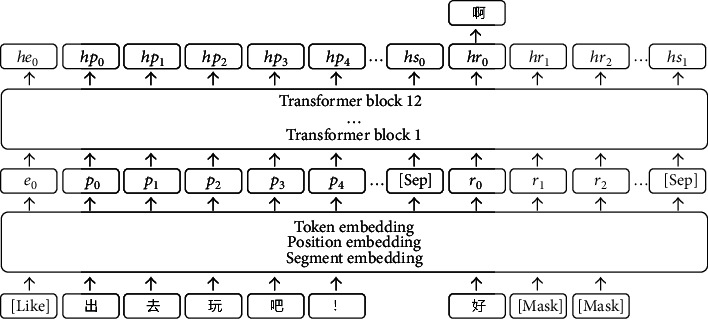
Overview of the CERG architecture. The input, from left to right, is emotion, post, and response. The model includes 3 embedding layers and 12 transformer blocks. The current position predicts the next character.

How to combine emotional computing with dialogue generation is a challenge. Zhou et al. proposed a memory-network-based emotional chatting machine [18], which introduced emotional factors into a Chinese dialogue generation system. We once proposed the P&E2R model based on the LSTM network [19]. On this basis, we build a new model to improve the effect of emotional response generation. Unlike our previous work, we use the same embedding layers to deal with the emotion, the post, and the response, as shown in Figure 1. Besides, the encoder and decoder are no longer established separately. We directly employ multiblock transformers, while masking the response part of the input text character by character to avoid information leakage. Based on the teacher-forcing method [20], we add regularization methods such as character replacement to alleviate the problems of overcorrection and exposure bias while ensuring the parallel training of the model. Apart from the beam search method, we employ the retrieval method to improve the semantic relevance of generated responses in inference.

This model has made great progress in the emotional response generation. The coherence, fluency, and emotional relevance scores of our model in manual evaluation are higher than those of the model without using the retrieval method and the baseline model. The proportion of safe and commonplace responses has also decreased significantly. These results indicate the effectiveness of our model. The model can be applied to the automatic reply of social applications like Chinese Weibo and emotional chatting robots.

Our contributions can be summarized as follows:

1. We propose a Chinese emotional response generator CERG, and the results on the Chinese Weibo dataset show that our model is effective. Without disrupting the parallel computing, we improve the robustness of the model by using the masking and regularization methods
2. We introduce the retrieval method BM25 into the inference process, which greatly reduces the probability of generating safe and commonplace responses and improves the diversity and contextual relevance of responses
3. We directly concatenate posts, mask responses with emotions, and adopt the embedding layers with shared weight to generate emotion-related answers, which is different from other models

The rest of this article is structured in the following part.  Section 2 briefly reviews the related work.  Section 3 provides the details of CERG.  Section 4 analyzes the experiments and the results of our model.  Section 5 presents the discussion, followed by the conclusion in Section 6.

## 2. Related Work

**Figure 2:**
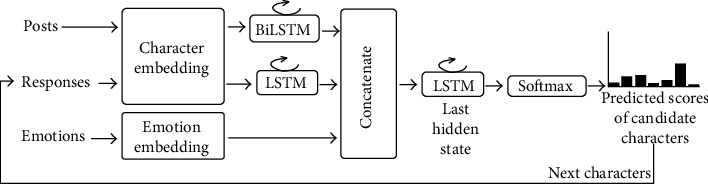
The baseline model from our previous work. Posts, responses, and emotions are concatenated by different encoding layers. The decoder is used to predict the next characters.

In the NTCIR-14 STC-3 CECG subtask, we proposed the P&E2R model and got the second rank, as shown in Figure 2. After embedding the posts and responses with a shared weighted layer, we encode them by the recurrent neural networks. The embedding emotions are concatenated with the former features. The probability distribution of the current word is generated by a recurrent neural network decoder. This model is simple but effective. We introduce the idea of concatenation in this article. The disadvantage of this model is that the calculation of the recurrent neural network depends on the hidden state of the previous time, and it cannot be parallelized, which is very time-consuming.

Dong et al. proposed the UniLM model [21]. The authors employ the transformer as the core of this model and make it parallel to improve calculation efficiency. Also, they adopt a special mask method to skillfully combine the encoder and decoder. Although we do not adopt the pretrained model from UniLM in our article, we introduce the idea of the attention mask method to improve the speed of the generator.

There are still some problems with this method. The teacher-forcing method is the key technology to ensure that the transformer model can completely calculate all tokens in parallel during the training process. Zhang et al. [22] pointed out that the ground truth word is used during model training, but once the predicted word is wrong in a certain position in the inference process, the output of the model will deviate from the predetermined direction. This will cause the exposure bias problem. The author proposed the word-level oracle and the sentence-level oracle method to solve the overcorrection problem brought by the teacher-forcing method. This method will disrupt the parallel computer system of the transformer model. We try to avoid disrupting the parallel computing mechanism and use a variety of regularization methods like predicted character replacement to make the model more robust.

In addition, we also employ a beam search method [23] in the inference process. Beam search is a search algorithm that explores a graph by expanding the most promising node in a limited set. On the basis of that, we use the BM25 method [24] and selects the most semantically relevant response among the *k* alternatives. BM25 is a ranking function to estimate the relevance of documents to a given search query. We adopt this method to find the responses of the *n* closest posts and calculate their similarity to the predicted responses. The experiments show that using this retrieval method can make the responses more in line with the context.

## 3. CERG Model

The emotional response generation task can be formulated as follows.

A post *P*_*i*_ = *p*_*i*0_, *p*_*i*1_, ⋯, *p*_*ik*_ and a kind of emotion *E*_*i*_, *E*_*i*_∈{“anger”, “disgust”, “happiness”, “like”, “sadness”}, are given. The goal is to predict a response *R*_*i*_ = *r*_*i*0_, *r*_*i*1_, ⋯, *r*_in_, (*r*_*i*0_, *r*_*i*1_, ⋯, *r*_in_ ∈ *C*). *C* is the character vocabulary of the texts.

We propose a model called CERG. As is illustrated in Figure 1, the core of this model is 12 transformer blocks. We take the emotion *E*_*i*_ and the post  *P*_*i*_ as the input. After initializing the parameters *θ* of the model *f* randomly, we concatenate the emotion *E*_*i*_, the post  *P*_*i*_, and the response *R*_*i*_ replaced by the “[Mask]” label in sequence. The sequence turns into the features after passing three embedding layers. The features are calculated by the transformer blocks and then turn into the hidden states. We try to train the model to minimize the cross-entropy loss function *l*_(*θ*)_ = −*Σ*_*r*_*j*_∈*w*_*r*_*j*_log*f*(*e*_0_, *p*_0_, ⋯, *p*_*L*−1_, *r*_0_, ⋯, *r*_*L*−1_; *θ*) . The process of backpropagation *θ* = *θ* − *η*(*∂l*_(*θ*)_/*∂θ*) makes *θ* approach the optimal value. When predicting, we adopt the hidden state where the first mask is located *h*_*r*_0__(*θ*) to predict the first character of the response *r*_1_. Then, replace the first mask with the first character *r*_1_ and continue to predict the second character  *r*_2_. Repeat the above process until the end symbol is predicted or the length of the response reaches the maximum length we set.

### 3.1. Baseline

We adopt the P&E2R model as the baseline in this article. There are a character embedding layer and an emotion embedding layer in this model. The posts and the responses share the weight through the character embedding layer. We encode the posts and responses separately by using two kinds of recurrent neural networks. The responses here are the predicted responses up to the last moment. The embedded emotions are concatenated with the hidden states of posts and responses. The decoder is another recurrent neural network. The decoding process is to predict the probability distribution of the next character based on the concatenated hidden states. This model achieved ranking second in manual evaluation.

### 3.2. Generator

As is shown in Figure 1, we put the emotion label in the first position, then concatenate it with the post and response. Unlike the baseline, emotion and text share the same embedding layers. The embedding layers consist of three parts. Token embedding is used to represent each character; position embedding is used to append the position of the character to the sentence; and segment embedding is used to distinguish between post and response. In the input text, we adopt the “[SEP]” label to separate the post and the response. We adopt the “[Mask]” label instead of the current predicted position and the position after it to prevent information leakage.

**Figure 3:**
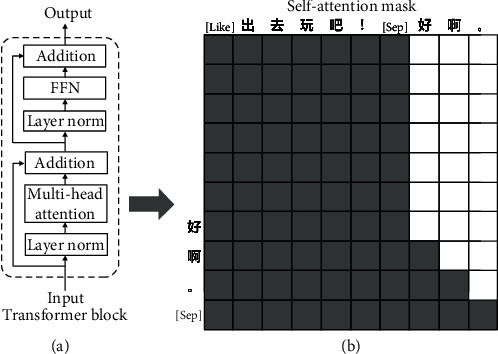
(a) Is the structure of the transformer block. (b) This matrix is an example of the self-attention mask.

A transformer is a framework in which attention structure replaces loop structure. The traditional transformer block consists of a multihead attention layer and a feedforward neural network (FFN) as the core.  Figure 3(a) shows that the layer normalization in each block is placed before the self-attention layer and the feed-forward layer. Xiong et al. [25] pointed out that placing layer normalization in this way can reduce the dependence of the model on the warm-up optimizer during training.

The attention matrix is shown on the right side of Figure 3. Unlike traditional transformers, we have to prevent the input response from leaking information to the output response. We employ teacher-forcing technology to expand an *n*−character response into *n* responses. During training, the output of the current character position will be the next character. As the example in Figure 3 shows, an “啊” would be generated in the hidden state of the position where “好” is located after training.

We also try to add some regularization methods to recover overcorrection without disrupting parallel computing. Before training, we adopt the language model BERT [26] to predict replacement characters at random positions in the input text. The replacement augmentation method can help to improve the robustness of the model [27]. In case the model is difficult to converge due to the use of regularization methods at the beginning of training, we sample the replacement characters with decay from the ground truth characters.

### 3.3. Retrieval Method

The retrieval method is applied in the inference process. We employ the beam search method to predict *n* responses. Then, we adopt the BM25 method to find *k* posts that are closest to the input post in the training set and calculate the similarity score *q*_0_, *q*_1_, ⋯, *q*_*k*−1_. Next, we calculate the similarity score  *a*_0,0_, *a*_0,1_, ⋯, *a*_0,*k*−1_ between the first predicted response and the corresponding responses of the *k* posts. The weighted score of the first response is  *a*_0_ = *a*_0,0_ × *q*_0_ + *a*_0,1_ × *q*_1_ + ⋯+*a*_0,*k*−1_ × *q*_*k*−1_. Similarly, the weighted score of the *n*th sentence is  *a*_*n*−1_ = *a*_*n*−1,0_ × *q*_0_ + *a*_*n*−1,1_ × *q*_1_ + ⋯+*a*_*n*−1,*k*−1_ × *q*_*k*−1_. Finally, we take the response with the highest weighted score as the output response. Experiments show that the general safe response cannot get high weighted scores here. This method can find out the responses that are more in line with the context of the posts and increase the diversity of the responses.

**Figure 4:**
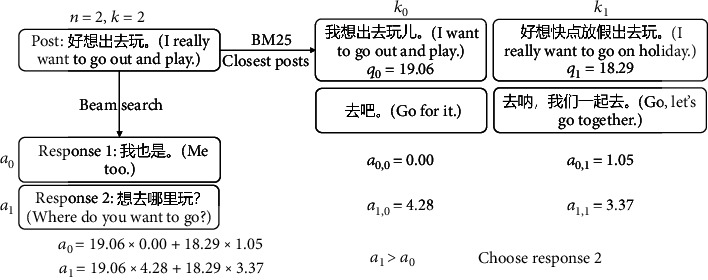
An example of using the retrieval method to select a better response in inference. The model predicts 2 candidate responses by the beam search method. The response with a lower beam score has a higher retrieval score.

For example, in Figure 4, we employ beam search (beam size = 2) to predict two responses on the left. We adopt the BM25 method to retrieve the two nearest posts from the training set. Then, we compare the similarity between the predicted responses and the corresponding responses of the retrieved posts. It can be seen from the comparison that response 2 with a lower score in beam search obtains a higher weighted score. We choose response 2 as the final result.

### 3.4. Model Setup

To balance efficiency and information loss, we set the maximum length of the posts and responses to 32. The size of the vocabulary is set to 13590. We set the embedding size and hidden size of the model to 768, which is consistent with the BERT-base model. We adopt 12 transformer blocks.

The training experiment shows that the larger the ratio of augmentation methods, the more difficult it is for the model to converge, and the time cost will also become larger. As the training epoch increases, we gradually increase the augmentation rate to 5%. We use NVIDIA 2080ti GPU training with batch size = 128. It takes about 2.3 hours to train an epoch.

The inference experiment shows that with the growth of the beam size and the retrieval *k*, the computational overhead becomes larger, but the improvement is not significant. The autoresponder needs to be timely. So we set these two parameters to 2.

## 4. Experiment and Evaluation

### 4.1. Data Set

The data set we adopt in this article comes from the NTCIR-14 STC-3 CECG task, which contains more than 1.7 million Chinese Weibo post-response pairs. The data set has already been tokenized. Because the size of the vocabulary is too large for the model training, we retokenize the texts into characters. According to our statistics, there are about 0.3% of the texts exceeding 32 characters in length. Considering the training efficiency and possible information loss, we set the length of the training texts to 32 characters.

Besides, we preprocess the texts. We check the data and find that there are some sentences without Chinese characters. We do not use these sentences for training. We also remove the extra duplicate characters and retain 3 times at most, for example, “哈哈哈.”

**Figure 5:**
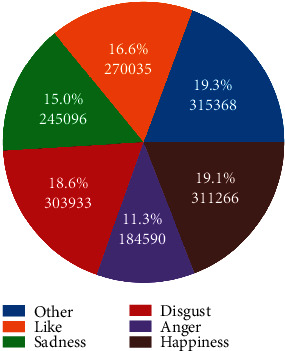
The distribution of different emotions in the data set.

There are 6 kinds of emotions in this dataset, including “anger,” “disgust,” “happiness,” “like,” “sadness,” and “other”. The emotion labels are classified on the real replies of Chinese Weibo by a classifier with an accuracy of about 64%, which are for reference only. We regard the imbalance in the number of categories as the noise of the data set. As can be seen from the pie chart in Figure 5, the “anger” category has the least amount of data. This may be one of the reasons for the worst performance of the “anger” category. The “other” item can help the model to generate smooth sentences during the training process, but this emotion is excluded during the inference process.

### 4.2. Evaluation Metrics

Consistent with the NTCIR-14 STC-3 task, we adopt 200 posts and 5 emotions to predict 1000 responses. Existing generation task automatic evaluation metrics such as BLEU [28] are not suitable for dialogue systems. For example, here is a post: “Someone injured.” According to the different contexts, “It is too pitiful” and “Who did it” are both reasonable responses. However, most of the automatic evaluation metrics calculate the similarity between the predicted sentence and the reference sentence through semantic or cooccurrence. We can find that not all reasonable responses can achieve high scores.

Therefore, the NTCIR-14 STC-3 task employs a manual evaluation method. If the predicted sentence is coherent and fluent, it can get the first point. On this basis, if the emotion of the sentence is consistent, it can get the second point. In this article, we adopt a similar but different scoring method. The deep learning generative models tend to predict safe and commonplace responses. In the experiment, we find that the reply using only emoji, “what's going on,” and “me too” are 3 main types of responses with a large number and often context-free. These 3 types of responses will not be scored in our evaluation process.  Table 1 is an example of our manual evaluation method.

Hard voting [29] is a commonly used ensemble method. We choose this method in manual evaluation. In addition, to verify the effectiveness of the retrieval method in our model, we made statistics and comparisons of the safe and commonplace responses.

### 4.3. Results

We compare the CERG model without the retrieval method and the full version of the CERG model with the baseline. The baseline results are taken from the responses we submitted to NTCIR-14. Tables 2–6 are the comparison results and the statistics about commonplace responses. The reason why the baseline gets lower scores than those published in NTCIR-14 is that we set all the safe and commonplace responses to label 0.

Table 2 shows the scores of the three models with the like emotion. The weighted average score of the model we proposed is 0.845, far exceeding the score at baseline. After we removed the retrieval method, our model also achieves a score of 0.575. Table 2 also shows the number of commonplace responses and their proportion in all responses. Nearly half of the responses generated at the baseline are emoji only. The emoji may express the respondents' emotions but has little to do with the context. The responses of the “me too” class in the no retrieval CERG model are more than those of the baseline. However, the proportions' of commonplace responses drop significantly in the complete CERG model.

Table 3 is about the sadness emotion. The increase in label 2 more likely comes from label 1, which is different from that of the like emotion. The proportion of commonplace responses is also less than that of the like emotion. The changes in the framework do not improve the result much, and the number of commonplace responses is similar. However, the use of the retrieval method increases the weighted average score to 0.735, and the number of commonplace responses decreases greatly as well.

Table 4 shows the experimental results of the disgust emotion. We can see that in the table, the generated responses are more coherent after we replace the framework. The emotional relevance of the responses also improves by using the retrieval method. Similar to the foregoing, the CERG model can reduce the proportion of commonplace responses in all the responses.

The experimental results of the anger emotion are shown in Table 5. The amount of training data of anger is the smallest. It might be the reason why the weighted average score of anger responses is lower than that of other emotions. Our model improves the average score to 0.625. There is no emoji in anger responses, and there are not many other commonplace responses. The CERG model still replaces most of these responses with more semantic and emotional responses.

Table 6 shows that there is a lot of emoji flood in happiness responses. We set responses containing the only emoji to label 0, so the score looks very low. Despite that, our CERG model raises the weighted average score to 0.755 and reduces the proportion of commonplace responses to 0.275.

## 5. Discussion

From the experimental results, we can conclude that the CERG model we proposed not only improves the speed of generating responses but also improves the textual representation ability, making the responses more coherent and fluent. On the basis of that, we also add the retrieval method to further improve the semantic relevance and emotional relevance of the responses. From our statistics on commonplace responses, the retrieval method can increase the diversity of responses and avoid context-free responses.

The CERG model maintains the parallelism of calculation while reducing the impact of exposure bias and overcorrection. During the experiment, using the retrieval method at the beginning would make the model difficult to converge. Besides, when the proportion of character replacement increases, the loss value decreases slowly. Therefore, we adopt the teacher-forcing method firstly and gradually replace part of the characters with the augmentation method. This can improve the robustness of the model.

Due to the training efficiency, the retrieval method we employ only focuses on a single character, rather than focusing on the whole word. We will improve this retrieval method in the next step, like optimizing the collocation between the current word and the previous word.

The anger emotion takes up the least proportion in the training data, which may be the reason why the evaluation score is not as high as other emotions. From the commonplace response analysis table, it can be seen that the response characteristics of each emotion are distinct. For example, the like emotion does not have “what's going on” responses, and the disgust emotion does not have “me too” responses. This may be related to the preference of the training data. It also shows that if we put the emotion label in the first item of text for input, the model can effectively distinguish different emotions.

There are more than these types of commonplace responses. We do not list other categories that are not typical. As can be seen from Figure 4, the keywords in posts rarely appear in commonplace responses. Therefore, we can easily reduce the weight of this type of response by using retrieval methods and sort more relevant responses before the commonplace response.

## 6. Conclusion

The emotional dialogue system has user-friendly human-computer interaction capabilities and can be applied to many domains such as psychotherapy. In this work, we propose the CERG model for Chinese Weibo emotional response generation. We combine the retrieval method with this generative model to improve the contextual relevance and diversity of generated responses.

The data we adopt comes from the NTCIR-14 STC-3 CECG subtask. The data set contains 6 emotion categories and the corresponding 1.7 million Chinese Weibo post-response pairs. After concatenating emotion, post, and response, we employ three embedding layers including token, position, and segment embedding layers and 12 transformer blocks for representation. To train the model with the conventional optimizer, we adjust the position of the layer normalization in the transformer blocks.

In the training process, we mask the response part of the input text character by character to emulate the encoder-decoder framework to prevent the leakage of information during inference. We replace the characters with the BERT model-predicted characters at random positions of the input text, which will improve the robustness of the model without disrupting the training parallelism. We introduce retrieval methods in the inference process. We calculate the weight scores of similar posts and responses together with beam search, which can make the predicted responses more in line with the context.

We adopt a hard voting manual metric to evaluate the generative ability of our model. The coherence, fluency, and emotional relevance scores of our model in the manual evaluation are higher than those of the model without the retrieval method and the baseline model. The proportion of safe and commonplace responses has also been greatly reduced. These results show the effectiveness of our model. The model can be applied to social applications like Chinese Weibo automatic reply robots.

In the next step, we will pay more attention to the combination of retrieval methods and word collocations to further reduce exposure bias due to the replacement we used. The code of the CERG model is available at https://github.com/youngzhou97qz/Beam-Search-Retrieval.

## Figures and Tables

**Table 1:** An example of manual evaluation.

Post	保佑我通过。(Bless me to pass.)	Emotion	Disgust
Response 1	我也是。(Me too.)	Label 0	Not coherent or not fluent or a safe response
Response 2	什么考试? (What exam?)	Label 1	Coherent and fluent
Response 3	你肯定挂科! (You will fail!)	Label 2	Coherent, fluent, and emotion consistent

**Table 2:** The evaluation result and the safe-response statistic of the like emotion.

Like	Label 0	Label 1	Label 2	Average	What's going on?	Me too	Only emoji	Proportion of safe responses
Baseline	142	53	5	0.315	0	10	95	0.525
No retrieval CERG	108	69	23	0.575	0	14	24	0.190
CERG	85	61	54	0.845	0	2	6	0.040

**Table 3:** The evaluation result and the safe-response statistic of the sadness emotion.

Sadness	Label 0	Label 1	Label 2	Average	What's going on?	Me too	Only emoji	Proportion of safe responses
Baseline	119	77	4	0.425	17	53	20	0.450
No retrieval CERG	104	83	13	0.545	4	27	56	0.435
CERG	99	55	46	0.735	1	9	7	0.085

**Table 4:** The evaluation result and the safe-response statistic of the disgust emotion.

Disgust	Label 0	Label 1	Label 2	Average	What's going on?	Me too	Only emoji	Proportion of safe responses
Baseline	132	67	1	0.345	54	0	19	0.365
No retrieval CERG	101	97	2	0.505	40	0	9	0.245
CERG	103	60	37	0.670	13	0	4	0.085

**Table 5:** The evaluation result and the safe-response statistic of the anger emotion.

Anger	Label 0	Label 1	Label 2	Average	What's going on?	Me too	Only emoji	Proportion of safe responses
Baseline	181	18	1	0.100	65	13	0	0.390
No retrieval CERG	135	59	6	0.355	16	37	0	0.265
CERG	92	91	17	0.625	1	4	0	0.025

**Table 6:** The evaluation result and the safe-response statistic of happiness emotion.

Happiness	Label 0	Label 1	Label 2	Average	What's going on?	Me too	Only emoji	Proportion of safe responses
Baseline	178	19	3	0.125	16	3	155	0.870
No retrieval CERG	140	49	11	0.335	10	11	108	0.645
CERG	85	79	36	0.755	6	2	47	0.275
